# Potential biomarkers of Ewing sarcoma identified through a Europe-wide analysis of prospectively collected samples

**DOI:** 10.1038/s41598-026-43071-0

**Published:** 2026-04-07

**Authors:** Andreas Ranft, Günther H. S. Richter, Juan Diaz-Martin, Susanne Jabar, Marvin Jens, Maximilian Kerkhoff, Maite Blanquer-Maceiras, Rosa Noguera, Inken Piro, Katja Steiger, Stefan Burdach, Alessandro Parra, Piero Picci, Marco Gambarotti, Wolfgang Hartmann, Katia Scotlandi, Enrique de Alava, Uta Dirksen

**Affiliations:** 1 Pediatrics III, University Hospital Essen, West German Cancer Center, Essen, Germany; 2 National Center for Tumor Diseases (NCT) Site Essen, German German Cancer Consortium (DKTK), Essen, Germany; 3 Department of Pediatrics, Division of Oncology and Hematology, Charite- Universitätsmedizin Berlin, Berlin, Germany; 4 German Cancer Consortium (DKTK), Partner Site Berlin, Berlin, Germany; 5 University of Valencia / INCLIVA / CIBERONC, Valencia, Spain; 6 Institute of Biomedicine of Sevilla, IBiS/Virgen del Rocio University Hospital, CSIC/University of Sevilla/CIBERONC, Seville, Spain; 7 Institute of Pathology, TUM School of Medicine and Health, Technische Universität München, Munich, Germany; 8 Department of Pediatrics and Children’s Cancer Research Center, TUM School of Medicine and Health, Technische Universität München; Zentrum für Kinder- und Jugendmedizin / Eine Kooperation der München Klinik und des TUM Klinikums, Munich, Germany; 9 Experimental Oncology Lab, IRCCS Istituto Ortopedico Rizzoli, Bologna, Italy; 10 Department of Pathology, IRCCS Istituto Ortopedico Rizzoli, Bologna, Italy; 11 Department of Pathology, University Hospital Muenster, Muenster, Germany; 12 Department of Normal and Pathological Cytology and Histology, School of Medicine, University of Seville, Seville, Spain

**Keywords:** Ewing sarcoma, Biomarkers, Prognosis, Biomarkers, Cancer, Genetics, Oncology

## Abstract

Clinical features, aside from disease stage, have proven insufficient to identify patients at extremely high or low risk. There are few established biological markers for EwS. The discovery of new prognostic and/or predictive biomarkers could improve our understanding of tumor heterogeneity, facilitate individual risk stratification, and aid targeted therapies. We initiated a pan-European study using formalin-fixed, prospectively collected, primary tumors prior to chemotherapy from 335 patients in two clinical trial registries, EURO-Ewing 99 and Ewing 2008, to analyze correlation and survival of ten potential prognostic biomarkers. Immunohistochemical (IHC) analyses were conducted for STEAP1, DKK2, and EZH2, while MIR34A and LGALS3BP expression was investigated by quantitative PCR. Genomic alterations, including gain of chromosome 1q, loss of ADAM3A, loss of chromosome 16q, as well as loss of heterozygosity (LOH) and percentage of the genome altered (PGA), were analyzed by FISH and/or SNP-based arrays of genomic DNA. To consider the observed variation in the number of analyzed samples per biomarker (N = 102–300), effect size measures were computed. Correlations between dichotomized biomarker expressions were generally low, except for those reflecting genomic alterations (ϕ >= 0.20 <= 0.38; *P *< .05). Several relevant correlations were found with clinical features of the underlying EwS patients (ϕ >= 0.20 <= 0.31; *P *< .05). Most promising prognostic values with clinically relevant effect sizes (OS), adjusted for known prognostic variables, were high MIR34A expression (HR = 0.29; *P *< .01), LOH (HR = 2.44; *P *< .05), and PGA (HR = 2.20; *P *= .05). This pan-European study identified MIR34A, LOH, and PGA as the most promising biomarkers for the prognosis of survival in a well-characterized EWS patient cohort.

**Supplementary Information:**

The online version contains supplementary material available at 10.1038/s41598-026-43071-0.

## Introduction

Ewing sarcoma (EwS) is a highly malignant tumor of bone and soft tissue that predominantly affects children and young adults, with a high propensity for early metastasis to the lungs and bones^1,2^. Although multimodal treatments have led to significant survival improvements in patients with localized disease, the prognosis for those with recurrent or metastatic disease remains poor, with a 5-year overall survival of less than 30%^3–5^. EwS is believed to arise from undifferentiated stem cells that exhibit features of both the mesenchymal and neural lineages. While individual genes in EwS are characterized by low mutation rates, the genome as a whole displays considerable cytogenetic aberrations and epigenetic heterogeneity^6–9^. EwS is defined by specific balanced chromosomal EWSR1::ETS translocations leading to chimeric proteins that function as aberrant transcription factors and determine the complex, and highly malignant phenotype^2,8^.

EwS exhibits substantial clinical heterogeneity^1^. Variability in EWSR1::ETS expression and transcriptional activity between individual tumor cells is a key factor in gene expression heterogeneity, tumor cell phenotype, and disease progression^10,11^. Several clinicopathological parameters, including tumor location, tumor volume, age at diagnosis, response to chemotherapy, and location of metastatic disease, have been identified as significant prognostic factors^12–15^. However, with the current international standard of compressed interval therapy, these clinical risk factors provide only limited prognostic information and have not been used to distinguish patients at particularly high or low risk for disease in risk-adapted therapeutic strategies^16^.

Currently, biological markers for EwS are limited. The identification of novel prognostic and/or predictive biomarkers could lead to a better understanding of tumor heterogeneity, enable individual risk stratification, and allow for targeted therapies^1,16^. In recent years, technological advances have enabled the investigation of various molecular biomarkers in EwS. Different EWSR1::ETS variants have been identified, with chromoplexy - a type of complex rearrangement - occurring in 42% of cases. Chromoplexy has been associated with increased relapse risk (54% vs. 30%, *P*<.05)^17^. Furthermore, EWS- tumors harboring ERG translocations show a variable histological spectrum^18^. However, these translocation variants have not yet yielded reliable biomarkers for individual risk stratification^2,12,19^.

Although EwS generally harbors a low mutational burden^8^, retrospective studies have identified copy number alterations (CNAs) that may be prognostically significant. Over 50% of EwS tumors present with non-random cytogenetic aberrations in addition to the characteristic EWSR1::ETS translocation^6^. In a retrospective study of 134 EwS patients, gain of chromosome (Chr.) 8 was observed in 52% of cases^6^. Some studies suggested a prognostic role for gains involving Chr. 8, including whole chromosome, segment 8q, MYC (8q24), and/or RAD21^20^. Chr. 8 gain^21^ and MYC amplification^22^ appear more frequent in recurrent tumors; however, larger cohort studies have not consistently confirmed a prognostic impact of Chr. 8 gains^6,23^. Gain of Chr. 12 was associated with worse event-free survival in patients with localized disease, while loss of 16q significantly correlated with poor overall survival and was linked to disseminated disease at diagnosis. Gain of Chr. 1q was associated with poor overall survival and event-free survival in all patients, independent of disease stage^6^. Furthermore, 1q gain, detected in 31% of EwS tumor samples, was strongly correlated with relapse and poor overall outcome irrespective of classical clinical risk parameters^24^.

Several deregulated genes in EwS also have the potential to serve as predictive markers. High protein expression of the membranous six-membrane epithelial antigen of the prostate 1 (STEAP1) has been associated with improved survival in a cohort of 114 chemotherapy-naive patients^25^. In contrast, high protein expression of the histone methyltransferase Enhancer of Zeste, Drosophila, homolog 2 (EZH2) was associated with poor survival in a study of 34 chemotherapy-naive patients^26,27^. mRNA and protein expression of lectin galactoside-binding soluble 3-binding protein (LGALS3BP) in EwS tumor tissues- but not in serum- was identified as a significant predictor of both event-free and overall survival, indicating a local role for the protein within the tumor microenvironment^28^. High expression of MIR34A has been associated with a more favorable clinical outcome, potentially due to increased sensitivity to chemotherapy in a cohort of 48 patients^29^, and its positive relationship with clinical outcome was further validated in specimens from 109 patients with localized, non-metastatic EwS treated with neoadjuvant chemotherapy^30^. Additionally, Dickkopf, Xenopus, homologue 2 (DKK2) protein expression, which is thought to mediate EwS-induced bone invasion via osteoclast activation and bone remodeling, correlated with decreased overall survival in a pilot study of 19 patients^31^.

These findings prompted us to initiate a pan-European study in a larger and prospective patient cohort to verify previous observations and validate the predictive and prognostic utility of selected biomarkers. Our study focuses on the following candidate biomarkers: LGALS3BP, MIR34A, loss of heterozygosity (LOH), ADAM3A, Chr 1q gain, Chr 16q loss, percentage of the genome altered (PGA), DKK2, EZH2, and STEAP1.

## Materials and methods

### Study population and sample collection

This study was part of a collaborative effort involving three European EwS study groups, forming the PROVABES consortium. This partnership facilitated the reliable collection of clinical data and biomaterial from newly diagnosed EwS patients across Europe. A uniform dataset of prospectively collected, well-characterized EwS cases with standardized clinical data was created. The work was conducted as an ancillary study to the Phase III clinical trials EWING 2008 (EudraCT 2008–003658-13, NCT00987636) and EURO-E.W.I.N.G 99 (NCT00020566; ISRCTN61438620). Detailed information on the trials can be found at clinicaltrialsregister.eu, clinicaltrials.gov, and controlled-trials.com. The trials were conducted in accordance with EU and national regulations and approved by the competent authorities. The patients’ consent forms for the trials included consent for ancillary studies such as PROVABES.

Patients included in the PROVABES analysis met the following criteria: registration in the European Phase III clinical trials participating in this consortium, inclusion and exclusion criteria according to the relevant protocol (including confirmed reference diagnosis of EwS), availability of biomaterials and informed consent for biomaterial collection and analysis. Registration of EwS patients in Phase III trials is population-based, as treatment in such trials is the standard of care for newly diagnosed patients. A total of 335 EwS samples were prospectively collected, with clinical data from the studies EE99 (66 samples) and EE2008 (269 samples). Data processing was performed in compliance with data protection regulations and overseen by an institutional review board. The international CESS study center was responsible for clinical data collection, processing, and analysis, using standardized operating procedures (SOPs). Tumor samples were collected prospectively from cooperating national and international biobanks (TranSaRNet, EuroBoNet) using PROVABES-specific SOPs. Formalin-fixed, paraffin-embedded (FFPE) tumor slides for genomic DNA extraction and tissue microarrays (TMA) for FISH probing or immunohistochemistry were provided to the respective biobanks by the reference pathologists from each study.

### Immunohistochemistry (IHC)

IHC analyses of DKK2, EZH2, and STEAP1 were performed on FFPE, chemotherapy-naive primary tumors. For IHC, blinded tissue microarrays (TMA) prepared from punch biopsies from the reference pathology of the EWING 2008 and EURO-E.W.I.N.G 99 study were obtained and stained in the Comparative Experimental Pathology, Department of Pathology, Technical University of Munich, using the Dako Autostainer system. After heat induced antigen retrieval (target retrieval solution, pH6, Dako, Glostrup, Denmark) for 20 min, unspecific protein and peroxidase binding was blocked with 3% hydrogen peroxide and 3% normal goat serum. The following primary antibodies were used: polyclonal rabbit anti-DKK2 (1:100; PAB3570; Abnova, Taipei, Taiwan), rabbit monoclonal anti-EZH2 (1:50; D2C9, Cell Signaling Technology, Danvers, MA, USA) and polyclonal rabbit anti-STEAP1 (1:50; H-105, sc-25514, Santa Cruz Biotechnology, Dallas, TX, USA). For antibody detection, for EZH2 and STEAP, the Dako Envision-HRP rabbit labeled polymer (Dako, Glostrup, Denmark) was used, for DKK2, a secondary biotinylated Goat-anti-Rabbit antibody (Vector Laboratories, Newark, CA, USA; BA-1000; 1:400) was used and antibody binding was detected with Strepatvidin Peroxidase (KPL immunologic, Gaithersburg, MD, USA). Antibody binding was visualized by diaminobenzidine (DAB) giving a brown precipitate (Medac Diagnostica, Wedel, Germany, BS04-500). Counterstaining was performed using hematoxylin. All sections were reviewed and interpreted by two pathologists (K.S. and I.P.).

### Quantitative PCR (qPCR)

Extraction from FFPE samples has been performed using ReliaPrep FFPE kit (Promega, Madison, WI, USA), following manufacturer instructions. All RNAs have been checked for quality and then reverse transcribed with Multiscribe Reverse Transcriptase kit (ThermoFisher Scientific, Waltham, MA, USA). A specific miRNA assay (hsa-mir-34a (#000426)) with corresponding control (RNU6-6P (#001093)) was used for MIR34A. A specific mRNA assay (Hs00174774_m1) and GAPDH (Hs99999905_m1) as a housekeeping gene were selected for the LGALS3BP gene product (all from ThermoFisher Scientific).

The qPCR has been performed on ABI7900HT Sequence Detection System apparatus qPCR system (Applied Biosystems) and the relative expression of the MIR34A and LGALS3BP were determined using the ddCt-RQ method using as calibrator RNA isolated from human mesenchymal stem cells (2 M-302, Lonza, Sciena, Italy), the putative cells of origin of EwS.

### Fluorescence in situ hybridization (FISH)

Tumor paraffin sections included in the TMAs were hybridized using the following commercial probes for FISH analyses: *ZytoLight* ^®^ SPEC MDM4/1p12 Dual Color Probe (ZytoVision, Bremerhaven, Germany) and ST 16qter (red)/ST 16pter (green) (KREATECH Diagnostics, Amsterdam, Netherlands). FISH results were evaluated and interpreted according to previously published procedures^32^. In brief, at least 100 nuclei/core were evaluated, ensuring the selection of non-overlapping nuclei and the analysis of multiple focal planes to ensure detection of all fluorescent signals. Control tissues (normal kidney, liver, and placenta) included in the TMAs were used to define the cutting effects of interphase FISH signals. We defined four groups: (1) cells without genetic modification with the same number of signals from the chromosome region (1q = 1p; 16q = 16p); (2) cells with possible gain of 1q and/or loss of 16q, with a different number of probe signals from the chromosome region 1q or 16q (targets) and control signals (1p or 16p); (3) cells with sectioning artifacts, including nuclear fragments with fewer target probe signals than in the control signals 1q < 1p and/or more 16q > 16p; (4) cells were confirmed 1q gain and/or 16q loss, calculated by subtracting the proportion of group 3 (cutting effects) from the cellular population of group 2. In control tissues, the mean + 3 standard deviations (SD) for group 4 did not exceed 15%. The same scoring system was applied to tumor samples, and a genetic alteration was considered present when more than 15% tumor fell in group 4. TMA were scored independently for each genetic marker by two authors (Bl-M M. and R N.).

### Single Nucleotide Polymorphism Microarray (SNPa)

FFPE 10 μm sections were used to isolate genomic DNA with QIAamp DNA FFPE Tissue Kit (Qiagen, Hilden, Germany). DNA concentration was determined using the Quanit-iT™ PicoGreen dsDNA assay (Invitrogen, Waltham, Massachusetts, USA) prior to genome-wide CNA analysis using molecular inversion probe SNP-based arrays (OncoScan FFPE Assay Kit, ThermoFisher Scientific), as previously described^33^. After scanning the arrays, CEL files were converted to OSCHP files with the TuScan algorithm, which estimates copy number changes based on B allele frequencies (BAFs) and log2 ratios, incorporating ploidy and aberrant cell percentages. Visualization of OSCHP files and population-level analyses of CNA and LOH were performed using Nexus Copy Number v10 software (Bionano Genomics). The percentage of the genome altered (PGA) and of LOH is computed by the software based on the proportion of the genome affected by CNAs and LOH respectively.

### Statistical analysis

A predefined power analysis had determined 335 localized samples would be needed to assess six biomarkers using a classical test design. However, due to a limited sample availability and the incremental inclusion of additional biomarkers during the study this design was deemed impractical. As a result, all analyses were conducted exploratively. No alpha correction was done for multiple testing. Accordingly, a *P*-value < .05 was interpreted only as a probabilistic indicator of statistical significance. Given the dependency of the *P*-values on sample size, and the wide variability in sample numbers per marker, the effect size measures were calculated^34,35^. These are less affected by sample size and offer a more robust assessment of clinical relevance. Pre-defined effect size conventions were applied to interpret clinical relevance.

The Fisher`s exact test was used to assess correlations of the dichotomized variables, and Phi(ϕ)-values were reported. Phi ranges from − 1 to 1, with values of 0.1 typically indicating small, 0.3 medium and 0.5 large effects, respectively. Some authors discuss lowering these thresholds to 0.1–0.2–0.3, as high effect sizes are rarely observed in the reality of human subject research^36^. Cox regression was used to calculate hazard ratios (HR) for survival as effect size measure. A HR of 2.0 or 0.5 was considered to define a strong clinically meaningful effect in EwS. Survival curves were calculated using the Kaplan-Meier estimates and compared using the log-rank test.

To summarize and prioritize findings, and to rebalance for the number of samples analyzed, a combined total score was developed to assess the exploratory validity of each biomarker, based on the effect size and *P*-value: 5 points were given each for HR >= 2 or <= 0.5 (|ϕ| >= 0.2) and *P *< .05; 2.5 points each for HR >= 1.5 to < 2 or <= 0.75 to > 0.5 (|ϕ| >= 0.1 to < 0.2) and *P* >= .05 to < .10. The summarized validity score (VS) resulted in 5 categories: 0, 2.5 (= X), 5 (= XX), 7.5 (= XXX), 10 (= XXXX), with 10 points representing the greatest evidence for the prognostic value of the underlying biomarker.

## Results

### Patient characteristics and samples

Patient samples were prospectively collected in two clinical trial registries that cover broadly similar treatment protocols over two decades: Euro-E.W.I.N.G. (EE) 99 (1998–2009), and Ewing 2008 (2009–2019). At the time of analysis, 335 samples were included in the study with 19.7% from EE 99 and 80.3% from Ewing 2008, (Table 1).

**Table 1:** Patient characteristics.

Characteristics	*N*	%
*Trial*	335	
EE99	66	19.7
Ewing 2008	269	80.3
*Age*	335	
< 15years	161	48.1
>=15years	174	51.9
*Sex*	335	
Male	190	56.7
Female	145	43.3
*Metastases Dx*	335	
No	230	68.7
Yes	105	31.3
*Tumor volume*	296	
< 200 ml	173	58.4
>=200 ml	123	41.6
*Pelvic location*	335	
No	268	80.0
Yes	67	20.0
*Histological response*	224	
Good	178	79.5
Poor	46	20.5
*Event*	335	
No	203	60.6
Yes	132	39.4
*Death*	335	
No	238	71.0
Yes	97	29.0

Most samples were evaluable for data on Chr. 1q gain and Chr. 16q loss and were analyzed by the Spanish Group. For LGALS3BP and MIR34A, which were analyzed by the Italian group, 102 of 335 samples were included in the analysis.

Chr. 1q gain, Chr. 16q loss and ADAM3A loss are binary features recorded as Yes/No events. Loss of ADAM3A, not previously reported in Ewing sarcoma, emerged in our SNP-array data as a recurrent microdeletion in the Chr. 8p11.22 region, present in 20.7% of analyzed cases. Therefore, despite not having been identified in prior studies, this focal CNA was included in the present validation study.

In contrast, PGA and LOH, MIR34A, LGALS3BP are continuous variables, which were dichotomized into high and low categories using the median as the cutoff. LOH was not specifically filtered for copy-neutral events during analysis. STEAP1, EZH2, and DKK2 were dichotomized using pre-defined classification values provided by the pathologists (Table 2).

**Table 2:** Available samples for biomarker expression analyses.

Biomarker	*N*	%
*LGALS3BP*	102	
Low	51	50.0
High	51	50.0
*MIR34A*	102	
Low	51	50.0
High	51	50.0
*LOH*	140	
Low	71	50.7
High	69	49.3
*PGA*	139	
Low	70	50.4
High	69	49.6
*ADAM3A*	140	
No	111	79.3
Yes	29	20.7
*Chr.1q gain*	300	
No	265	88.3
Yes	35	11.7
*Chr.16q loss*	278	
No	245	88.1
Yes	33	11.9
*EZH2*	234	
Low	185	79.1
High	49	20.9
*DKK2*	212	
Low	188	88.7
High	24	11.3
*STEAP1*	238	
Low	147	61.8
High	91	38.2

### Correlations with clinical parameters

Biomarkers were correlated with dichotomized clinical variables and prognostic factors such as age (< 15; >= 15 years), sex (male; female), metastases at diagnosis (no; yes), primary tumor volume (< 200 ml; >= 200 ml), pelvic location (no; yes), histological response (< 10%; >= 10% viable cells), events (no; yes), and death (no; yes).

For all results see Table 3 and Supplementary Tables 4–14. Meaningful results with a high exploratory validity score (>= 7.5 points) are listed here:

#### STEAP1

High STEAP1 expression (*N* = 91 of 238) was correlated with fewer observed deaths (20.9% vs. 34.0%; ϕ = −0.14; *P *= .039; VS = 7.5).

#### EZH2

Patients with high EZH2 expression (*N* = 49 of 234) were more likely to have metastases at diagnosis (51.0% versus 26.5%; ϕ = 0.22; *P*=.002; VS = 10).

#### Chromosome 1q gain

The occurrence of Chr. 1q gain (*N* = 33 of 267) was correlated with more pelvic primaries (36.4% versus 17.6%; ϕ = 0.19; *P *= .018; VS = 7.5).

#### PGA

A high PGA (*N* = 69 of 139) was correlated to older age (81.2% versus 64.3%; ϕ = 0.19; *P *< .036; VS = 7.5), more pelvic primaries (36.2% versus 10.0%; ϕ = 0.31; *P *< .001), a poor histological response (24.4% versus 7.7%; ϕ = 0.23; *P *= .027), more events (53.6% versus 28.6%; ϕ = 0.26; *P *= .003), and more deaths (37.7% versus 15.7%; ϕ = 0.25; *P *= .004) (VS = 10).

#### LOH

Patients with high LOH expression (*N* = 69 of 140) were more likely to have pelvic primaries (36.2% versus 11.3%; ϕ = 0.29; *P *< .001), and more observed deaths (36.2% versus 16.9%; ϕ = 0.22; *P *= .012) (VS = 10).

#### MIR34A

A high expression of MIR34A (*N* = 51 of 102) was correlated with less frequency in females (37.3% versus 56.9%; ϕ = −0.20; *P *= .074), fewer observed events (33.3% versus 52.9%; ϕ = −0.20; *P *= .071) (VS = 7.5), and fewer observed deaths (17.6% versus 41.2%; ϕ = −0.26; *P *= .016; VS = 10).

The highest effect sizes (ϕ >= 0.20) in this cohort were observed across multiple rates for PGA, LOH, and MIR34A, as well as in the correlation between EZH2 and metastatic disease. All of them reached *P *< .05,* except for* MIR34A due to a limited number of samples. While DKK2 expression, Chromosome 16q loss, ADAM3A loss, and LGALS3BP expression were not highly associated with clinical parameters (VS <= 2.5).

### Correlations between biomarkers

Most correlations between biomarkers were low (see the correlation matrix in Supplementary Table 15).

Moderate to relatively large correlations with VS = 7.5 were observed for STEAP1 and LGALS3BP (ϕ = −0.23; *P*= .061), and with VS = 10 for DKK2 and EZH2 (ϕ = 0.20; *P *= .009), Chr. 16q and Chr. 1q (ϕ = 0.27; *P *< .001), Chr. 16q and PGA (ϕ = 0.33; *P *< .001), Chr. 16q and LOH (ϕ = 0.38; *P *< .001), Chr. 1q and PGA (ϕ = 0.24; *P *= .004), Chr. 1q and LOH (ϕ = 0.20; *P *< .001), and PGA and LOH (ϕ = 0.37; *P *< .001).

**Table 3:** Effect size Phi (ϕ); *P >= .05 < .10; **=*P *< .05 and validity score (VS): 0; X = 2.5; XX = 5; XXX = 7.5; XXXX = 10.

		STEAP1	DKK2	EZH2	Chr. 16q	Chr. 1q	PGA	ADAM3A	LOH	MIR34A	LGALS3BP
	*N*	238	212	234	278	300	140	140	140	102	102
Age	ϕ	0.05	−0.01	−0.12*	−0.08	−0.02	0.19**	0.07	0.09	0.04	0.00
	VS	0	0	XX	0	0	XXX	0	0	0	0
Sex	ϕ	−0.05	−0.06	−0.03	−0.02	0.02	−0.05	−0.07	−0.00	−0.20*	0.00
	VS	0	0	0	0	0	0	0	0	XXX	0
Metastases Dx	ϕ	0.03	−0.12	0.22**	0.06	0.04	0.07	−0.08	0.07	−0.02	0.11
	VS	0	X	XXXX	0	0	0	0	0	0	X
Tumor volume	ϕ	−0.10	−0.01	0.04	0.07	0.10	−0.01	0.11	0.00	0.00	−0.09
	VS	X	0	0	0	X	0	X	0	0	0
Pelvic location	ϕ	−0.09	0.01	−0.02	0.05	0.19**	0.31**	0.01	0.29**	0.10	0.10
	VS	0	0	0	0	XXX	XXXX	0	XXXX	X	X
Hist. response	ϕ	−0.09	0.15	0.01	0.07	−0.00	0.23**	−0.01	−0.05	0.09	−0.01
	VS	0	X	0	0	0	XXXX	0	0	0	0
Event	ϕ	−0.12*	0.04	−0.00	0.04	0.10*	0.26**	−0.00	0.16*	−0.20*	0.12
	VS	XX	0	0	0	XX	XXXX	0	XX	XXX	X
Death	ϕ	−0.14**	−0.05	0.04	0.09	0.11*	0.25**	−0.03	0.22**	−0.26**	0.00
	VS	XXX	0	0	0	XX	XXXX	0	XXXX	XXXX	0

### Survival

#### Hazard ratios

**Fig. 1:**
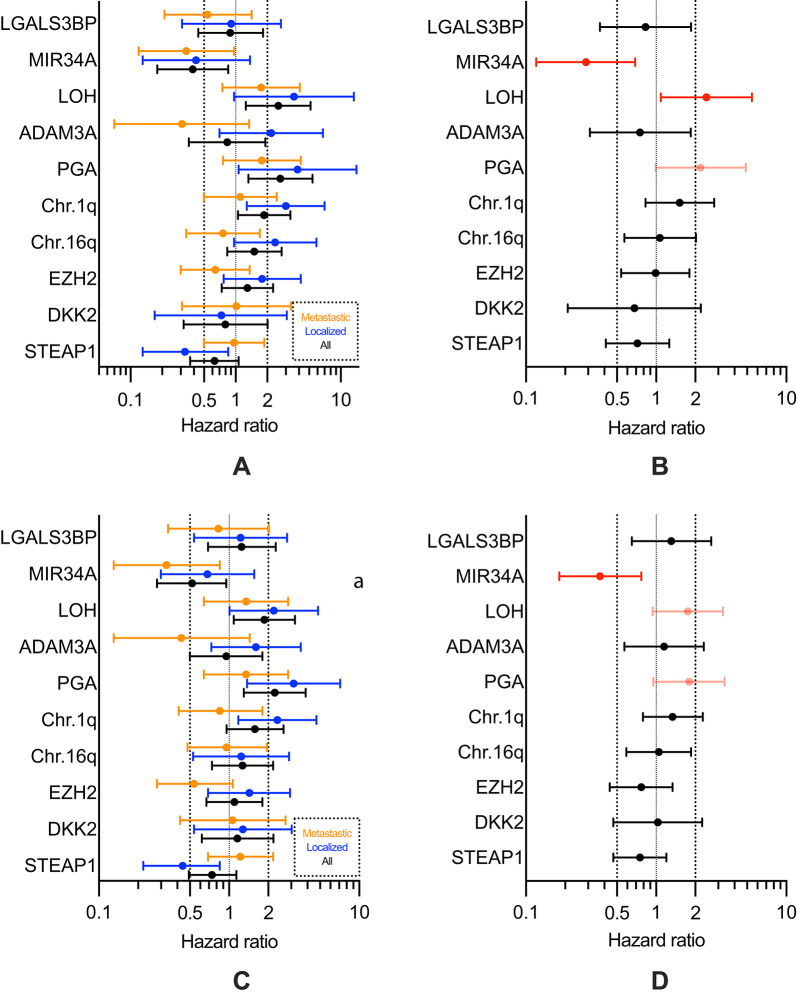
Unadjusted overall survival (OS) (**A**); event-free survival (EFS) (**C**), and adjusted OS (**B**) and EFS (**D**) (in dark red validity score (VS) = 10; in light red VS = 5–7.5.5), effect sizes (HR and 95% CI) for high expression of biomarkers.

Unadjusted HR for all patients, and for localized and metastatic disease separately, are shown in Fig. 1 A (OS), 1 C (EFS), and adjusted HRs corrected for sex (male vs. female), age (< 15 years vs. >= 15 years), site (pelvic vs. non-pelvic), volume (< 200 ml vs. >= 200 ml), disease status (localized vs. metastatic) in Fig. 1B (OS), 1D (EFS).

##### Unadjusted HR (Supplementary Table 16)

In all patients, at least a doubled (or halved) HR for high expression of biomarkers were observed for PGA (OS: HR = 2.67; EFS: HR = 2.24), LOH (OS: HR = 2.55), and MIR34A (OS: HR = 0.39) (*P *< .05; VS = 10).

For the subgroup of localized patients, at least a doubled (or halved) HR were observed for STEAP1 (OS: HR = 0.33; EFS: HR = 0.44), Chr.1q (OS: HR = 3.00; EFS: HR = 2.35), PGA (OS: HR = 3.89; EFS: HR = 3.14), LOH (EFS: HR = 2.21) (*P *< .05; VS = 10), Ch.16q (OS: HR = 2.38), LOH (OS: HR = 3.60; *P *< .10) (VS = 7.5), MIR34A (OS: HR = 0.42) and ADAM3A (OS: HR = 2.18) (*P *> .10; VS = 5).

In metastatic patients, at least a doubled (or halved) HR were observed for MIR34A (OS: HR = 0.34; EFS: HR = 0.33; *P *< .05; VS = 10), and ADAM3A (OS: HR = 0.31; EFS: HR = 0.43; *P *> .10; VS = 5).

##### Adjusted HR (Supplementary Table 17)

At least a doubled (or halved) HR for high expression of biomarkers, were observed for MIR34A (OS: HR = 0.29; EFS: HR = 0.37), LOH (OS: HR = 2.44) (*P *< .05; VS = 10) and PGA (OS: HR = 2.20; *P *< .10; VS = 7.5).

#### Kaplan-Meier estimates

Baseline of all patients was 0.78 (SE = 0.02) in 3y-OS, and 0.67 (SE = 0.03) in 3y-EFS.

**Fig. 2:**
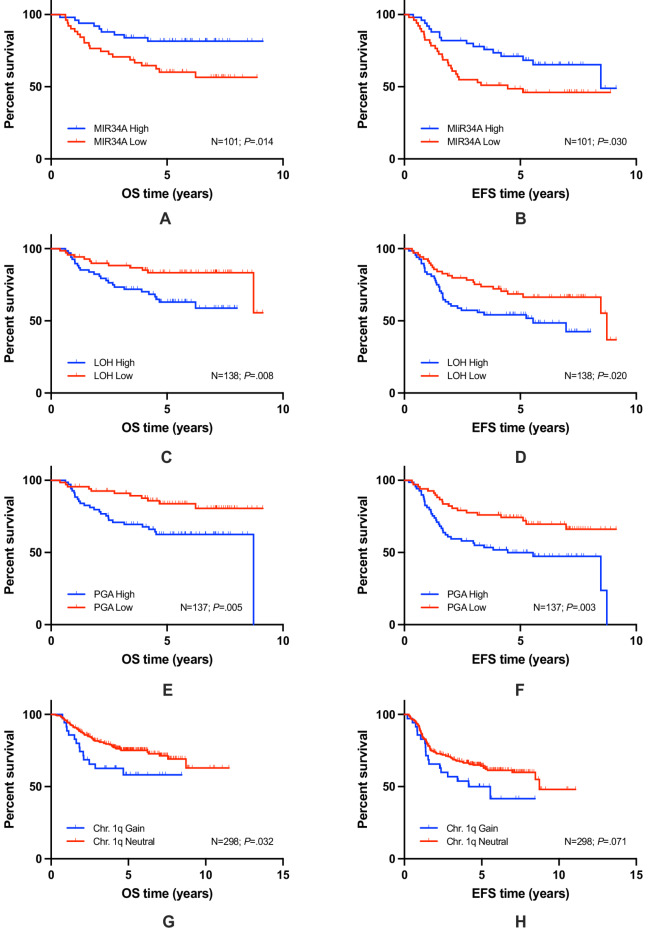
Kaplan-Meier plots for individual biomarkers are given and results for overall (OS) and event-free (EFS) survival are shown. OS/EFS of MIR34A (**A**, **B**), LOH (**C**, **D**), PGA (**E**, **F**), Chr. 1q (**G**, **H**) (N: number of patients investigated; *P*: log-rank comparison).

Highest Delta (Δ) in 3y-OS in Kaplan-Meier (KM) log-rank comparisons with *P *< .05 was achieved in PGA (Fig. 2E) of 0.20 (SE = 0.01) followed by Chr.1q (Fig. 2G) of 0.19 (SE = 0.01), MIR34A (Fig. 2-A) of 0.15 (SE = 0.01), and LOH (Fig. 2C) of 0.15 (SE = 0.01).

**Fig. 3:**
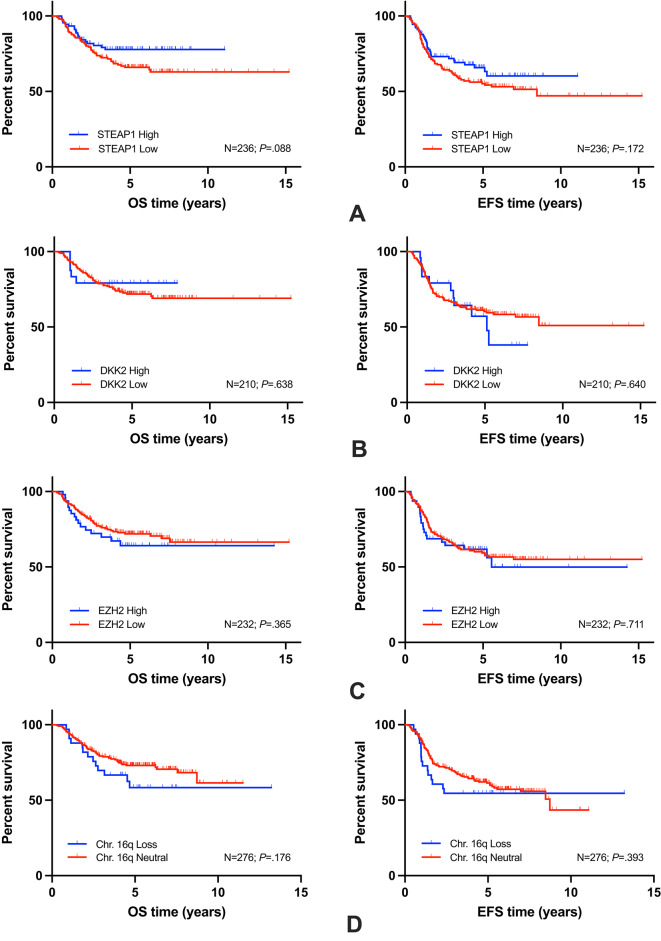

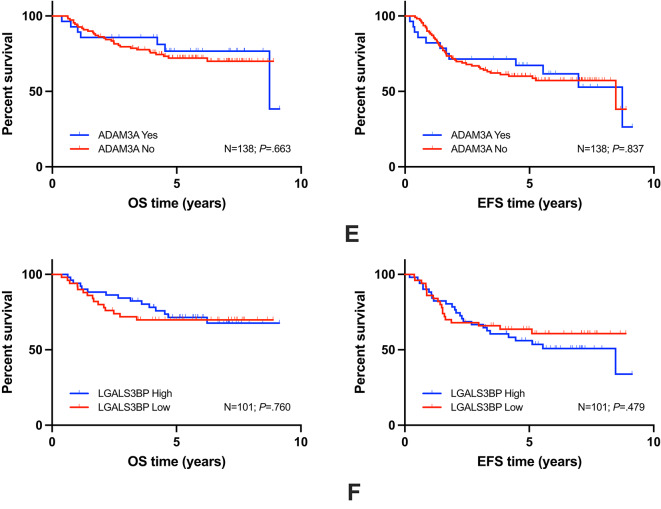
Kaplan-Meier plots for individual biomarkers are given and results for overall (OS) and event-free (EFS) survival are shown: OS/EFS of STEAP1 (**A**), DKK2 (**B**), EZH2 (**C**), Chr. 16q (**D**), ADAM3A loss (**E**), LGALS3BP (**F**) (N: number of patients investigated; *P*: log-rank comparison).

Highest Δ in 3y-EFS in KM log-rank comparisons with *P *< .05 was observed for MIR34A (Fig. 2B) of 0.29 (SE = 0.01), followed by PGA (Fig. 2F) of 0.21 (SE = 0.01), and LOH (Fig. 2D) of 0.19 (SE = 0.01). Other markers with KM log-rank comparisons with *P* >= 0.05 are listed in Fig. 3.

## Discussion

Currently, disease status is the most informative clinical marker for prognosis in EwS, but neither in patients with localized disease nor within the collective of metastatic and/or relapsed patients can individuals with possible differential response to therapy be predicted or identified^13,14,16^. Prognostic biomarkers in EwS to predict the risk of progression or recurrence have not been clinically validated^1^. To investigate potential biomarkers, we therefore pooled data from biological samples analysis of three large European EwS study groups as part of the PROVABES consortium. We created a uniform dataset of well-characterized EwS cases with homogeneous clinical data. Ten potential biomarkers were analyzed in 335 prospectively collected EwS samples. LOH, PGA and increased MIR34A were identified as prognostic biomarkers in EwS. Likewise, we recently reported the prospective validation of chr. 1q as a prognostic biomarker in patients with localized disease^37^.

A prognostic value could not be confirmed for most of the deregulated genes with MIR34A, and STEAP1 in localized patients, forming exceptions. One reason for the lack of significance could be the plasticity of the expression profile of these genes. Modulation of EWS::FLI1-dependent gene signatures, without altering the expression of the fusion itself, influences the state of tumor cells^38^ and presumably contributes to spatial variations of EwS cells in the tumor region^38,39^. Differential expression of tissue-specific transcription factors^40,41^, the modulation of the epigenetic profile^26,42^ and other cellular and cell-external mechanisms^11^ additionally influence the transcriptional activity of EWS::FLI1 and may affect its expression profile and overall tumorigenic and metastatic properties of EwS cells. These factors modulate the stability of expression of individual deregulated genes repeatedly observed in EwS and limit their potential as prognostic biomarkers.

However, in our study population, high membranous STEAP1 expression was still associated with a good prognosis in patients with localized disease, as has already been shown in a smaller cohort of 114 patients^25^. At the molecular level, high expression of STEAP1 is associated with elevated intracellular ROS and may improve accessibility to chemotherapeutic agents^25,43^. Investigating the strength of STEAP1 expression, therefore, remains an important biomarker for EFS or OS in patients with localized disease.

In addition, low EZH2 expression was found to be associated with metastatic disease but not with OS. Mechanistic data previously indicated that a high EZH2 expression correlated with stemness and malignancy of EwS^26,27^. Further investigations are required to better understand how low EZH2 expression correlates with EwS metastasis.

Interestingly, in this study population, consistent with previous findings^29,30^, MIR34A appears to play a role in suppressing tumors. Expression of MIR34A was found to be decreased in many cancers^44^. In experimental cancer models, re-expression of MIR34A was found to induce cell-cycle arrest, apoptosis, chemosensitivity, while counteracting cell migration, epithelial mesenchymal transition and metastasis through the regulation of a plethora of targets, including E2F, cyclin D1, CDK4, CDK6, cyclin E2, Bcl-2, β-catenin, Notch, MDM4^45^ and was identified as an important regulator of immune and inflammatory responses in cancer, indicating a multifaceted role for this miRNA. In EwS, reduced expression in tumor samples was associated with higher cyclin D1 and Ki-67 expression, cancer treatment resistance, and worse prognosis^29,30^. In addition, the detection of MIR34A in the blood of patients with EwS was previously correlated with tumor volume^46^. In this study, we further confirm the clinical relevance of low MIR34A expression as a biomarker of EwS patients’ prognosis, supporting its detection either in tumors and/or in blood to predict therapeutic responses and disease progression.

Structural chromosomal changes are frequently found in EwS, including gain of Chr. 1q, 2, 8, and 12, and losses of 9p and 16q^47–49^. Since arm-length aneuploidies involve broad genomic regions containing numerous genes, their oncogenic impact may stem from the cumulative action of multiple interacting genetic elements rather than a single driver^50,51^.

In the current study, chromosomal Chr. 1q gain and Chr. 16q loss were examined in a larger and prospectively collected group of EwS patient samples. Remarkably, as we recently reported, the large study population revealed that Chr. 1q gain was associated with negative survival outcomes in patients with localized disease, but not in metastatic patients^37^. Another recent study analyzing a retrospective cohort of 196 EwS cases confirmed the clinical impact of Chr. 1q gain, although multivariate analyses did not support its independence from clinical variables^52^. In contrast, the latest Children’s Oncology Group (COG) study on localized EwS patients enrolled in frontline COG trials did not identify any association between individual CNAs and patient outcome, even in univariate analyses^53^. Methodological differences in CNA assessment may account for these discrepancies.

In parallel, a CNA profile was generated for some of the patient samples using SNPa to evaluate the feasibility of these arrays as a clinical determination and to prospectively evaluate the influence of the genome-wide CNA profile on patient prognosis. We analyzed the genome-wide CNA profile in a subset of 140 samples with available material for DNA preparation. SNPa analyses showed that gain events were much more frequent than losses, identifying recurrent CNA patterns consistent with previous studies^7,23,24,48^.

We and others have described that the extent/number of CNAs or increased PGA appear to be predictor of prognosis, being consistently associated with poor outcome in EwS and a gradual decrease in survival^7,23,24,48,54–56^. Here, we examined the EwS cases with available CNA profile observing that high PGA correlates with poor histologic response and pelvic localization (Supplementary Table 10). Furthermore, as we recently reported, PGA emerged as strong independent prognostic factor along with metastatic disease^37^.

LOH was also determined via SNP arrays and was identified as a strong predictor of clinical outcomes of the investigated patients. LOH and copy-neutral LOH are two mechanisms by which tumors can inactivate tumor suppressor genes often achieving the classic “two-hit” inactivation required for loss of function. It is noteworthy that Ewing sarcomas are characterized by low mutation rates^8^, yet show genomic alterations in a high number of cases, which manifest as chromosomal gains and losses, representing independent biomarkers for EwS. It is not yet clear which functional mechanisms underlie this chromosomal instability. However, there are indications of possible causes^57^. For example, the formation of EWSR1 fusion genes in EwS leads to the loss of one or both wild-type EWSR1 alleles in the sarcoma cells^58,59^. This loss can lead directly to LOH, as demonstrated in fish models^60^, and it has been hypothesized that the involvement of wild-type EWSR1 in chromosome segregation may be a possible cause^60,61^. However, the exact biological mechanisms that lead to LOH in Ewing sarcomas and determine its extent need to be investigated further.

Our results show that it is essential to analyze genomic alterations in Ewing patients as PGA or LOH are independent prognostic markers for disease progression. The recent switch to interval-compressed chemotherapy as the standard treatment^62^ could influence either the prognostic validity of previously identified clinical prognostic factors or the biomarkers newly derived from this study with the former non-compressed treatment. Further validation studies using samples based on this new standard treatment could therefore be beneficial in the future. However, a deviation is unlikely, as the clinical factors known to date have been considered stable for the development of treatments for decades, the underlying chemotherapeutic agents have not changed fundamentally, and the samples in this study were taken before treatment. It needs to be further investigated whether additional genomic markers, such as chromoplexy, as an alternative mechanism to simple reciprocal translocation observed not only in EwS but also in other sarcomas associated with translocations^17,63^, allow the definition of a combined genomic variation index that further improves the observed results on LOH and PGA.

In conclusion, this pan-European study has identified high LOH and PGA, and low MIR34A expression as the most relevant biomarkers for poor EFS and OS in a well-characterized cohort of patients with EwS.

## Supplementary Information

Below is the link to the electronic supplementary material.

Supplementary Information.

## Data Availability

The data are used under license and are not publicly available. Data are however available from the authors upon reasonable request and with permission of the sponsor of the trial.
