# Supplementary material for: Potential biomarkers of Ewing sarcoma identified through a Europe-wide analysis of prospectively collected samples
Source: Sci Rep. 2026 Apr 7;16:11613. doi: 10.1038/s41598-026-43071-0 (PMC13057395; doi:10.1038/s41598-026-43071-0)
Supplement: Supplementary file 1 — Supplementary Information. [file 41598_2026_43071_MOESM1_ESM.docx]

**Supplementary Information**

# Supplementary Table 4. Patient characteristics / Trial

|  |  | *Trial* | | |  |  |
| --- | --- | --- | --- | --- | --- | --- |
|  |  | *EE 99* |  | *Ewing 2008* |  |  |
| *Characteristics* | N | N | % | N | % | *P* |
| *Age* | 335 |  |  |  |  | <.001 |
| *<15years* | 161 | 48 | 72.7 | 113 | 42.0 |  |
| *>=15years* | 174 | 18 | 27.3 | 156 | 58.0 |  |
|  |  |  |  |  |  |  |
| *Sex* | 335 |  |  |  |  | .680 |
| *Male* | 190 | 39 | 59.1 | 151 | 56.1 |  |
| *Female* | 145 | 27 | 40.9 | 118 | 43.9 |  |
|  |  |  |  |  |  |  |
| *Metastases Dx* | 335 |  |  |  |  | .236 |
| *No* | 230 | 41 | 62.1 | 189 | 70.3 |  |
| *Yes* | 105 | 25 | 37.9 | 80 | 29.7 |  |
|  |  |  |  |  |  |  |
| *Tumor volume* | 296 |  |  |  |  | .775 |
| *<200ml* | 173 | 36 | 56.25 | 137 | 59.1 |  |
| *>=200ml* | 123 | 28 | 43.75 | 95 | 40.9 |  |
|  |  |  |  |  |  |  |
| *Pelvic location* | 335 |  |  |  |  | 1.00 |
| *No* | 268 | 53 | 80.3 | 215 | 79.9 |  |
| *Yes* | 67 | 13 | 19.7 | 54 | 20.1 |  |
|  |  |  |  |  |  |  |
| *Hist. response* | 224 |  |  |  |  | 1.00 |
| *Good* | 178 | 28 | 80.0 | 150 | 79.4 |  |
| *Poor* | 46 | 7 | 20.0 | 39 | 20.6 |  |
|  |  |  |  |  |  |  |
| *Event* | 335 |  |  |  |  | .265 |
| *No* | 203 | 36 | 54.5 | 167 | 62.1 |  |
| *Yes* | 132 | 30 | 45.5 | 102 | 37.9 |  |
|  |  |  |  |  |  |  |
| *Death* | 335 |  |  |  |  | .095 |
| *No* | 238 | 41 | 62.1 | 197 | 73.2 |  |
| *Yes* | 97 | 25 | 37.9 | 72 | 26.8 |  |

#

# Supplementary Table 5. Patient characteristics / STEAP1

|  |  | *STEAP1* | | |  |  |
| --- | --- | --- | --- | --- | --- | --- |
|  |  | *Low* |  | *High* |  |  |
| *Characteristics* | N | N | % | N | % | *P* |
| *Age* | 238 |  |  |  |  | .503 |
| *<15years* | 107 | 69 | 46.9 | 38 | 41.8 |  |
| *>=15years* | 131 | 78 | 53.1 | 53 | 58.2 |  |
|  |  |  |  |  |  |  |
| *Sex* | 238 |  |  |  |  | .503 |
| *Male* | 131 | 78 | 53.1 | 53 | 58.2 |  |
| *Female* | 107 | 69 | 46.9 | 38 | 41.8 |  |
|  |  |  |  |  |  |  |
| *Metastases Dx* | 238 |  |  |  |  | .671 |
| *No* | 161 | 101 | 68.7 | 60 | 65.9 |  |
| *Yes* | 77 | 46 | 31.3 | 31 | 34.1 |  |
|  |  |  |  |  |  |  |
| *Tumor volume* | 204 |  |  |  |  | .185 |
| *<200ml* | 123 | 71 | 56.3 | 52 | 66.7 |  |
| *>=200ml* | 81 | 55 | 43.7 | 26 | 33.3 |  |
|  |  |  |  |  |  |  |
| *Pelvic location* | 238 |  |  |  |  | .241 |
| *No* | 191 | 114 | 77.6 | 77 | 84.6 |  |
| *Yes* | 47 | 33 | 22.4 | 14 | 15.4 |  |
|  |  |  |  |  |  |  |
| *Hist. response* | 158 |  |  |  |  | .315 |
| *Good* | 124 | 79 | 76.0 | 45 | 83.3 |  |
| *Poor* | 34 | 25 | 24.0 | 9 | 16.7 |  |
|  |  |  |  |  |  |  |
| *Event* | 238 |  |  |  |  | .079 |
| *No* | 139 | 79 | 53.7 | 60 | 65.9 |  |
| *Yes* | 99 | 68 | 46.3 | 31 | 34.1 |  |
|  |  |  |  |  |  |  |
| *Death* | 238 |  |  |  |  | .039 |
| *No* | 169 | 97 | 66.0 | 72 | 79.1 |  |
| *Yes* | 69 | 50 | 34.0 | 19 | 20.9 |  |

#

# Supplementary Table 6. Patient characteristics / DKK2

|  |  | *DKK2* | | |  |  |
| --- | --- | --- | --- | --- | --- | --- |
|  |  | *Low* |  | *High* |  |  |
| *Characteristics* | N | N | % | N | % | *P* |
| *Age* | 212 |  |  |  |  | 1.00 |
| *<15years* | 86 | 76 | 40.4 | 10 | 41.7 |  |
| *>=15years* | 126 | 112 | 59.6 | 14 | 58.3 |  |
|  |  |  |  |  |  |  |
| *Sex* | 212 |  |  |  |  | .515 |
| *Male* | 115 | 100 | 53.2 | 15 | 62.5 |  |
| *Female* | 97 | 88 | 46.8 | 9 | 37.5 |  |
|  |  |  |  |  |  |  |
| *Metastases Dx* | 212 |  |  |  |  | 1.00 |
| *No* | 147 | 130 | 69.1 | 17 | 70.8 |  |
| *Yes* | 65 | 58 | 30.9 | 7 | 29.2 |  |
|  |  |  |  |  |  |  |
| *Tumor volume* | 180 |  |  |  |  | 1.00 |
| *<200ml* | 108 | 97 | 59.9 | 11 | 61.1 |  |
| *>=200ml* | 72 | 65 | 40.1 | 7 | 38.9 |  |
|  |  |  |  |  |  |  |
| *Pelvic location* | 212 |  |  |  |  | 1.00 |
| *No* | 170 | 151 | 80.3 | 19 | 79.2 |  |
| *Yes* | 42 | 37 | 19.7 | 5 | 20.8 |  |
|  |  |  |  |  |  |  |
| *Hist. response* | 146 |  |  |  |  | .127 |
| *Good* | 115 | 103 | 81.1 | 12 | 63.2 |  |
| *Poor* | 31 | 24 | 18.9 | 7 | 36.8 |  |
|  |  |  |  |  |  |  |
| *Event* | 212 |  |  |  |  | .663 |
| *No* | 125 | 112 | 59.6 | 13 | 54.2 |  |
| *Yes* | 87 | 76 | 40.4 | 11 | 45.8 |  |
|  |  |  |  |  |  |  |
| *Death* | 212 |  |  |  |  | .627 |
| *No* | 155 | 136 | 72.3 | 19 | 79.2 |  |
| *Yes* | 57 | 52 | 27.7 | 5 | 20.8 |  |

#

# Supplementary Table 7. Patient characteristics / EZH2

|  |  | *EZH2* | | |  |  |
| --- | --- | --- | --- | --- | --- | --- |
|  |  | *Low* |  | *High* |  |  |
| *Characteristics* | N | N | % | N | % | *P* |
| *Age* | 234 |  |  |  |  | .076 |
| *<15years* | 106 | 78 | 42.2 | 28 | 57.1 |  |
| *>=15years* | 128 | 107 | 57.8 | 21 | 42.9 |  |
|  |  |  |  |  |  |  |
| *Sex* | 234 |  |  |  |  | .748 |
| *Male* | 128 | 100 | 54.1 | 28 | 57.1 |  |
| *Female* | 106 | 85 | 45.9 | 21 | 42.9 |  |
|  |  |  |  |  |  |  |
| *Metastases Dx* | 234 |  |  |  |  | .002 |
| *No* | 160 | 136 | 73.5 | 24 | 49.0 |  |
| *Yes* | 74 | 49 | 26.5 | 25 | 51.0 |  |
|  |  |  |  |  |  |  |
| *Tumor volume* | 202 |  |  |  |  | .610 |
| *<200ml* | 117 | 93 | 58.9 | 24 | 54.5 |  |
| *>=200ml* | 85 | 65 | 41.1 | 20 | 45.5 |  |
|  |  |  |  |  |  |  |
| *Pelvic location* | 234 |  |  |  |  | .842 |
| *No* | 187 | 147 | 79.5 | 40 | 81.6 |  |
| *Yes* | 47 | 38 | 20.5 | 9 | 18.4 |  |
|  |  |  |  |  |  |  |
| *Hist. response* | 155 |  |  |  |  | 1.00 |
| *Good* | 125 | 97 | 80.8 | 28 | 80.0 |  |
| *Poor* | 30 | 23 | 19.2 | 7 | 20.0 |  |
|  |  |  |  |  |  |  |
| *Event* | 234 |  |  |  |  | 1.00 |
| *No* | 138 | 109 | 58.9 | 29 | 59.2 |  |
| *Yes* | 96 | 76 | 41.1 | 20 | 40.8 |  |
|  |  |  |  |  |  |  |
| *Death* | 234 |  |  |  |  | .596 |
| *No* | 166 | 133 | 71.9 | 33 | 67.3 |  |
| *Yes* | 68 | 52 | 28.1 | 16 | 32.7 |  |

#

# Supplementary Table 8. Patient characteristics / Chr. 16q

|  |  | *Chr. 16q* | | |  |  |
| --- | --- | --- | --- | --- | --- | --- |
|  |  | *Neutral* |  | *Loss* |  |  |
| *Characteristics* | N | N | % | N | % | *P* |
| *Age* | 278 |  |  |  |  | .195 |
| *<15years* | 129 | 110 | 44.9 | 19 | 57.6 |  |
| *>=15years* | 149 | 135 | 55.1 | 14 | 42.4 |  |
|  |  |  |  |  |  |  |
| *Sex* | 278 |  |  |  |  | .852 |
| *Male* | 160 | 140 | 57.1 | 20 | 60.6 |  |
| *Female* | 118 | 105 | 42.9 | 13 | 39.4 |  |
|  |  |  |  |  |  |  |
| *Metastases Dx* | 278 |  |  |  |  | .329 |
| *No* | 183 | 164 | 66.9 | 19 | 57.6 |  |
| *Yes* | 95 | 81 | 33.1 | 14 | 42.4 |  |
|  |  |  |  |  |  |  |
| *Tumor volume* | 244 |  |  |  |  | .335 |
| *<200ml* | 139 | 124 | 58.2 | 15 | 48.4 |  |
| *>=200ml* | 105 | 89 | 41.8 | 16 | 51.6 |  |
|  |  |  |  |  |  |  |
| *Pelvic location* | 278 |  |  |  |  | .376 |
| *No* | 218 | 194 | 79.2 | 24 | 72.8 |  |
| *Yes* | 60 | 51 | 20.8 | 9 | 27.2 |  |
|  |  |  |  |  |  |  |
| *Hist. response* | 181 |  |  |  |  | .390 |
| *Good* | 147 | 130 | 82.3 | 17 | 73.9 |  |
| *Poor* | 34 | 28 | 17.7 | 6 | 26.1 |  |
|  |  |  |  |  |  |  |
| *Event* | 278 |  |  |  |  | .573 |
| *No* | 166 | 148 | 60.4 | 18 | 54.5 |  |
| *Yes* | 112 | 97 | 39.6 | 15 | 45.5 |  |
|  |  |  |  |  |  |  |
| *Death* | 278 |  |  |  |  | .148 |
| *No* | 200 | 180 | 73.5 | 20 | 60.6 |  |
| *Yes* | 78 | 65 | 26.5 | 13 | 49.4 |  |

#

# Supplementary Table 9. Patient characteristics / Chr. 1q

|  |  | *Chr. 1q* | | |  |  |
| --- | --- | --- | --- | --- | --- | --- |
|  |  | *Neutral* |  | *Gain* |  |  |
| *Characteristics* | N | N | % | N | % | *P* |
| *Age* | 300 |  |  |  |  | .857 |
| *<15years* | 139 | 122 | 46.0 | 17 | 48.6 |  |
| *>=15years* | 161 | 143 | 54.0 | 18 | 51.4 |  |
|  |  |  |  |  |  |  |
| *Sex* | 300 |  |  |  |  | .721 |
| *Male* | 171 | 152 | 57.4 | 19 | 54.3 |  |
| *Female* | 129 | 113 | 42.6 | 16 | 45.7 |  |
|  |  |  |  |  |  |  |
| *Metastases Dx* | 300 |  |  |  |  | .563 |
| *No* | 204 | 182 | 68.7 | 22 | 62.9 |  |
| *Yes* | 96 | 83 | 31.3 | 13 | 37.1 |  |
|  |  |  |  |  |  |  |
| *Tumor volume* | 262 |  |  |  |  | .129 |
| *<200ml* | 154 | 139 | 60.7 | 15 | 45.5 |  |
| *>=200ml* | 108 | 90 | 39.3 | 18 | 54.5 |  |
|  |  |  |  |  |  |  |
| *Pelvic location* | 300 |  |  |  |  | .003 |
| *No* | 241 | 220 | 83.0 | 21 | 60.0 |  |
| *Yes* | 59 | 45 | 17.0 | 14 | 40.0 |  |
|  |  |  |  |  |  |  |
| *Hist. response* | 204 |  |  |  |  | 1.00 |
| *Good* | 163 | 147 | 79.9 | 16 | 80.0 |  |
| *Poor* | 41 | 37 | 20.1 | 4 | 20.0 |  |
|  |  |  |  |  |  |  |
| *Event* | 300 |  |  |  |  | .099 |
| *No* | 185 | 168 | 63.4 | 17 | 48.6 |  |
| *Yes* | 115 | 97 | 36.6 | 18 | 51.4 |  |
|  |  |  |  |  |  |  |
| *Death* | 300 |  |  |  |  | .068 |
| *No* | 220 | 199 | 75.1 | 21 | 60.0 |  |
| *Yes* | 80 | 66 | 24.9 | 14 | 40.0 |  |

#

# Supplementary Table 10. Patient characteristics / PGA

|  |  | *PGA* | | |  |  |
| --- | --- | --- | --- | --- | --- | --- |
|  |  | *Low* |  | *High* |  |  |
| *Characteristics* | N | N | % | N | % | *P* |
| *Age* | 139 |  |  |  |  | .036 |
| *<15years* | 38 | 25 | 35.7 | 13 | 18.8 |  |
| *>=15years* | 101 | 45 | 64.3 | 56 | 81.2 |  |
|  |  |  |  |  |  |  |
| *Sex* | 139 |  |  |  |  | .605 |
| *Male* | 83 | 40 | 57.1 | 43 | 62.3 |  |
| *Female* | 56 | 30 | 42.9 | 26 | 37.7 |  |
|  |  |  |  |  |  |  |
| *Metastases Dx* | 139 |  |  |  |  | .469 |
| *No* | 95 | 50 | 71.4 | 45 | 65.2 |  |
| *Yes* | 44 | 20 | 28.5 | 24 | 34.8 |  |
|  |  |  |  |  |  |  |
| *Tumor volume* | 115 |  |  |  |  | 1.00 |
| *<200ml* | 66 | 33 | 56.9 | 33 | 57.9 |  |
| *>=200ml* | 49 | 25 | 43.1 | 24 | 42.1 |  |
|  |  |  |  |  |  |  |
| *Pelvic location* | 139 |  |  |  |  | <.001 |
| *No* | 107 | 63 | 90.0 | 44 | 63.8 |  |
| *Yes* | 32 | 7 | 10.0 | 25 | 36.2 |  |
|  |  |  |  |  |  |  |
| *Hist. response* | 97 |  |  |  |  | .027 |
| *Good* | 82 | 48 | 92.3 | 34 | 75.6 |  |
| *Poor* | 15 | 4 | 7.7 | 11 | 24.4 |  |
|  |  |  |  |  |  |  |
| *Event* | 139 |  |  |  |  | .003 |
| *No* | 82 | 50 | 71.4 | 32 | 46.4 |  |
| *Yes* | 57 | 20 | 28.6 | 37 | 53.6 |  |
|  |  |  |  |  |  |  |
| *Death* | 139 |  |  |  |  | .004 |
| *No* | 102 | 59 | 84.3 | 43 | 62.3 |  |
| *Yes* | 37 | 11 | 15.7 | 26 | 37.7 |  |

#

# Supplementary Table 11. Patient characteristics / ADAM3A

|  |  | *ADAM3A* | | |  |  |
| --- | --- | --- | --- | --- | --- | --- |
|  |  | *No* |  | *Yes* |  |  |
| *Characteristics* | N | N | % | N | % | *P* |
| *Age* | 140 |  |  |  |  | .485 |
| *<15years* | 38 | 32 | 28.8 | 6 | 20.7 |  |
| *>=15years* | 102 | 79 | 71.2 | 23 | 79.3 |  |
|  |  |  |  |  |  |  |
| *Sex* | 140 |  |  |  |  | .527 |
| *Male* | 83 | 64 | 57.7 | 19 | 65.5 |  |
| *Female* | 57 | 47 | 42.3 | 10 | 34.5 |  |
|  |  |  |  |  |  |  |
| *Metastases Dx* | 140 |  |  |  |  | .379 |
| *No* | 96 | 74 | 66.7 | 22 | 75.9 |  |
| *Yes* | 44 | 37 | 33.3 | 7 | 24.1 |  |
|  |  |  |  |  |  |  |
| *Tumor volume* | 116 |  |  |  |  | .252 |
| *<200ml* | 66 | 55 | 59.8 | 11 | 45.8 |  |
| *>=200ml* | 50 | 37 | 40.2 | 13 | 54.2 |  |
|  |  |  |  |  |  |  |
| *Pelvic location* | 140 |  |  |  |  | 1.00 |
| *No* | 107 | 85 | 76.6 | 22 | 75.9 |  |
| *Yes* | 33 | 26 | 23.4 | 7 | 24.1 |  |
|  |  |  |  |  |  |  |
| *Hist. response* | 97 |  |  |  |  | 1.00 |
| *Good* | 82 | 65 | 84.4 | 17 | 85.0 |  |
| *Poor* | 15 | 12 | 15.6 | 3 | 15.0 |  |
|  |  |  |  |  |  |  |
| *Event* | 140 |  |  |  |  | 1.00 |
| *No* | 82 | 65 | 58.6 | 17 | 58.6 |  |
| *Yes* | 58 | 46 | 41.4 | 12 | 41.4 |  |
|  |  |  |  |  |  |  |
| *Death* | 140 |  |  |  |  | .818 |
| *No* | 103 | 81 | 73.0 | 22 | 75.9 |  |
| *Yes* | 37 | 30 | 27.0 | 7 | 24.1 |  |

#

# Supplementary Table 12. Patient characteristics / LOH

|  |  | *LOH* | | |  |  |
| --- | --- | --- | --- | --- | --- | --- |
|  |  | *Low* |  | *High* |  |  |
| *Characteristics* | N | N | % | N | % | *P* |
| *Age* | 140 |  |  |  |  | .345 |
| *<15years* | 38 | 22 | 31.0 | 16 | 23.1 |  |
| *>=15years* | 102 | 49 | 69.0 | 53 | 76.8 |  |
|  |  |  |  |  |  |  |
| *Sex* | 140 |  |  |  |  | 1.00 |
| *Male* | 83 | 42 | 59.2 | 41 | 59.4 |  |
| *Female* | 57 | 29 | 40.8 | 28 | 40.6 |  |
|  |  |  |  |  |  |  |
| *Metastases Dx* | 140 |  |  |  |  | .468 |
| *No* | 96 | 51 | 71.8 | 45 | 65.2 |  |
| *Yes* | 44 | 20 | 28.2 | 24 | 34.8 |  |
|  |  |  |  |  |  |  |
| *Tumor volume* | 116 |  |  |  |  | 1.00 |
| *<200ml* | 66 | 33 | 56.9 | 33 | 56.9 |  |
| *>=200ml* | 50 | 25 | 43.1 | 25 | 43.1 |  |
|  |  |  |  |  |  |  |
| *Pelvic location* | 140 |  |  |  |  | <.001 |
| *No* | 107 | 63 | 88.7 | 44 | 63.8 |  |
| *Yes* | 33 | 8 | 11.3 | 25 | 36.2 |  |
|  |  |  |  |  |  |  |
| *Hist. response* | 97 |  |  |  |  | .781 |
| *Good* | 82 | 44 | 83.0 | 38 | 86.4 |  |
| *Poor* | 15 | 9 | 17.0 | 6 | 13.6 |  |
|  |  |  |  |  |  |  |
| *Event* | 140 |  |  |  |  | .086 |
| *No* | 82 | 47 | 66.2 | 35 | 50.7 |  |
| *Yes* | 58 | 24 | 33.8 | 34 | 49.3 |  |
|  |  |  |  |  |  |  |
| *Death* | 140 |  |  |  |  | .012 |
| *No* | 103 | 59 | 83.1 | 44 | 63.8 |  |
| *Yes* | 37 | 12 | 16.9 | 25 | 36.2 |  |

#

# Supplementary Table 13. Patient characteristics / MIR34A

|  |  | *MIR34A* | | |  |  |
| --- | --- | --- | --- | --- | --- | --- |
|  |  | *Low* |  | *High* |  |  |
| *Characteristics* | N | N | % | N | % | *P* |
| *Age* | 102 |  |  |  |  | .825 |
| *<15years* | 28 | 15 | 29.4 | 13 | 25.5 |  |
| *>=15years* | 74 | 36 | 70.6 | 38 | 74.5 |  |
|  |  |  |  |  |  |  |
| *Sex* | 102 |  |  |  |  | .074 |
| *Male* | 54 | 22 | 43.1 | 32 | 62.7 |  |
| *Female* | 48 | 29 | 56.9 | 19 | 37.3 |  |
|  |  |  |  |  |  |  |
| *Metastases Dx* | 102 |  |  |  |  | 1.00 |
| *No* | 73 | 36 | 70.6 | 37 | 72.5 |  |
| *Yes* | 29 | 15 | 29.4 | 14 | 27.5 |  |
|  |  |  |  |  |  |  |
| *Tumor volume* | 86 |  |  |  |  | 1.00 |
| *<200ml* | 44 | 22 | 51.2 | 22 | 51.2 |  |
| *>=200ml* | 42 | 21 | 48.6 | 21 | 48.6 |  |
|  |  |  |  |  |  |  |
| *Pelvic location* | 102 |  |  |  |  | .471 |
| *No* | 80 | 42 | 82.4 | 38 | 74.5 |  |
| *Yes* | 22 | 9 | 17.6 | 13 | 25.5 |  |
|  |  |  |  |  |  |  |
| *Hist. response* | 73 |  |  |  |  | .556 |
| *Good* | 59 | 32 | 84.2 | 27 | 77.1 |  |
| *Poor* | 14 | 6 | 15.8 | 8 | 22.9 |  |
|  |  |  |  |  |  |  |
| *Event* | 102 |  |  |  |  | .071 |
| *No* | 58 | 24 | 47.1 | 34 | 66.7 |  |
| *Yes* | 44 | 27 | 52.9 | 17 | 33.3 |  |
|  |  |  |  |  |  |  |
| *Death* | 102 |  |  |  |  | .016 |
| *No* | 72 | 30 | 58.8 | 42 | 82.4 |  |
| *Yes* | 30 | 21 | 41.2 | 9 | 17.6 |  |

#

# Supplementary Table 14. Patient characteristics / LGALS3BP

|  |  | *LGALS3BP* | | |  |  |
| --- | --- | --- | --- | --- | --- | --- |
|  |  | *Low* |  | *High* |  |  |
| *Characteristics* | N | N | % | N | % | *P* |
| *Age* | 102 |  |  |  |  | 1.00 |
| *<15years* | 28 | 14 | 27.5 | 14 | 27.5 |  |
| *>=15years* | 74 | 37 | 72.5 | 37 | 72.5 |  |
|  |  |  |  |  |  |  |
| *Sex* | 102 |  |  |  |  | 1.00 |
| *Male* | 54 | 27 | 52.9 | 27 | 52.9 |  |
| *Female* | 48 | 24 | 47.1 | 24 | 47.1 |  |
|  |  |  |  |  |  |  |
| *Metastases Dx* | 102 |  |  |  |  | .380 |
| *No* | 73 | 39 | 76.5 | 34 | 66.7 |  |
| *Yes* | 29 | 12 | 23.5 | 17 | 33.3 |  |
|  |  |  |  |  |  |  |
| *Tumor volume* | 86 |  |  |  |  | .516 |
| *<200ml* | 44 | 18 | 46.2 | 26 | 55.3 |  |
| *>=200ml* | 42 | 21 | 53.8 | 21 | 44.7 |  |
|  |  |  |  |  |  |  |
| *Pelvic location* | 102 |  |  |  |  | .471 |
| *No* | 80 | 42 | 82.4 | 38 | 74.5 |  |
| *Yes* | 22 | 9 | 17.6 | 13 | 25.5 |  |
|  |  |  |  |  |  |  |
| *Hist. response* | 73 |  |  |  |  | 1.00 |
| *Good* | 59 | 29 | 80.6 | 30 | 81.1 |  |
| *Poor* | 14 | 7 | 19.4 | 7 | 18.9 |  |
|  |  |  |  |  |  |  |
| *Event* | 102 |  |  |  |  | .318 |
| *No* | 58 | 32 | 62.7 | 26 | 51.0 |  |
| *Yes* | 44 | 19 | 37.3 | 25 | 49.0 |  |
|  |  |  |  |  |  |  |
| *Death* | 102 |  |  |  |  | 1.00 |
| *No* | 72 | 36 | 70.6 | 36 | 70.6 |  |
| *Yes* | 30 | 15 | 29.4 | 15 | 29.4 |  |

Supplementary Table 15: Correlations of biomarkers: Phi (top right) & *P*-Values (left bottom)

|  | STEAP1 | DKK2 | EZH2 | Chr. 16q | Chr. 1q | PGA | ADAM3A | LOH | MIR34A | LGALS3BP |
| --- | --- | --- | --- | --- | --- | --- | --- | --- | --- | --- |
| STEAP1 |  | 0.10 | 0.11 | -0.12 | -0.07 | -0.11 | -0.03 | -0.16 | -0.09 | **-0.23** |
| DKK2 | .187 |  | **0.20** | 0.04 | 0.05 | 0.05 | 0.10 | -0.07 | 0.06 | -0.19 |
| EZH2 | .121 | .009 |  | 0.05 | 0.05 | 0.14 | 0.03 | 0.05 | -0.01 | -0.04 |
| Chr. 16q | .157 | .706 | .579 |  | **0.27** | **0.33** | 0.08 | **0.38** | -0.08 | -0.00 |
| Chr. 1q | .394 | .503 | .611 | <.001 |  | **0.24** | 0.03 | **0.20** | -0.09 | -0.03 |
| PGA | .338 | .770 | .213 | <.001 | .004 |  | 0.02 | **0.37** | -0.09 | -0.11 |
| ADAM3A | .818 | .461 | .767 | .525 | 1.00 | .837 |  | 0.06 | -0.01 | 0.09 |
| LOH | .126 | .564 | .628 | <.001 | .020 | <.001 | .535 |  | 0.01 | -0.08 |
| MIR34A | .488 | .739 | 1.00 | .540 | .554 | .521 | 1.00 | 1.00 |  | 0.02 |
| LGALS3BP | .061 | .097 | .762 | 1.00 | 1.00 | .394 | .430 | .527 | 1.00 |  |

# Supplementary Table 16. Hazard ratios (EFS, OS); *P*-value and validity score (VS): 0; X=2.5; XX=5; XXX=7.5; XXXX=10; loc=localized; met=metastatic

|  |  | STEAP1 | DKK2 | EZH2 | Chr. 16q | Chr. 1q | PGA | ADAM3A | LOH | MIR34A | LGALS3BP |
| --- | --- | --- | --- | --- | --- | --- | --- | --- | --- | --- | --- |
| EFS | HR | 0.74 | 1.16 | 1.10 | 1.27 | 1.58 | 2.24 | 0.95 | 1.87 | 0.52 | 1.25 |
|  | *P* | .173 | .641 | .706 | .394 | .074 | .004 | .863 | .022 | .034 | .463 |
|  | VS | X | 0 | 0 | 0 | XX | XXXX | 0 | XXX | XXX | 0 |
| OS | HR | 0.63 | 0.80 | 1.30 | 1.51 | 1.86 | 2.67 | 0.83 | 2.55 | 0.39 | 0.89 |
|  | *P* | .090 | .640 | .366 | .179 | .035 | .006 | .663 | .010 | .018 | .760 |
|  | VS | XX | 0 | 0 | X | XXX | XXXX | 0 | XXXX | XXXX | 0 |
| EFS loc | HR | 0.44 | 1.28 | 1.43 | 1.24 | 2.35 | 3.14 | 1.61 | 2.21 | 0.68 | 1.23 |
|  | *P* | .009 | .590 | .338 | .627 | .015 | .006 | .239 | .047 | .368 | .615 |
|  | VS | XXXX | 0 | 0 | 0 | XXXX | XXXX | X | XXXX | X | 0 |
| OS loc | HR | 0.33 | 0.73 | 1.79 | 2.38 | 3.00 | 3.89 | 2.18 | 3.60 | 0.42 | 0.91 |
|  | *P* | .021 | .663 | .174 | .058 | .023 | .024 | .180 | .055 | .150 | .861 |
|  | VS | XXXX | X | X | XXX | XXXX | XXXX | XX | XXX | XX | 0 |
| EFS met | HR | 1.22 | 1.06 | 0.54 | 0.96 | 0.85 | 1.35 | 0.43 | 1.35 | 0.33 | 0.83 |
|  | *P* | .493 | .896 | .078 | .918 | .678 | .427 | .172 | .427 | .021 | .682 |
|  | VS | 0 | 0 | XX | 0 | 0 | 0 | XX | 0 | XXXX | 0 |
| OS met | HR | 0.97 | 1.02 | 0.64 | 0.76 | 1.11 | 1.77 | 0.31 | 1.75 | 0.34 | 0.54 |
|  | *P* | .923 | .968 | .248 | .502 | .802 | .188 | .117 | .198 | .043 | .209 |
|  | VS | 0 | 0 | X | 0 | 0 | X | XX | X | XXXX | X |

# Supplementary Table 17. Adjusted hazard ratios (HR); *P*-value and validity score (VS): 0; X=2.5; XX=5; XXX=7.5; XXXX=10

|  |  | STEAP1 | DKK2 | EZH2 | Chr. 16q | Chr. 1q | PGA | ADAM3A | LOH | MIR34A | LGALS3BP |
| --- | --- | --- | --- | --- | --- | --- | --- | --- | --- | --- | --- |
| EFS | HR | 0.75 | 1.03 | 0.77 | 1.05 | 1.34 | 1.79 | 1.15 | 1.75 | 0.37 | 1.32 |
|  | *P* | .224 | .943 | .354 | .878 | .274 | .072 | .707 | .078 | .008 | .443 |
|  | VS | X | 0 | 0 | 0 | 0 | XX | 0 | XX | XXXX | 0 |
| OS | HR | 0.72 | 0.68 | 0.99 | 1.07 | 1.52 | 2.20 | 0.75 | 2.44 | 0.29 | 0.83 |
|  | *P* | .250 | .525 | .961 | .843 | .177 | .053 | .533 | .029 | .005 | .653 |
|  | VS | X | X | 0 | 0 | X | XXX | X | XXXX | XXXX | 0 |
